# A core outcome set for clinical trials of Chinese medicine for hyperlipidemia: a study protocol for a systematic review and a Delphi survey

**DOI:** 10.1186/s13063-018-3082-9

**Published:** 2019-01-07

**Authors:** Geng Li, Li Zhou, Wenwei Ouyang, Meiling Xuan, Liming Lu, Xiaoyan Li, Zehuai Wen, Xiankun Chen

**Affiliations:** 1 Mathematical Engineering Academy of Chinese Medicine, Guangzhou University of Chinese Medicine, Guangzhou, Guangdong China; 2 The Second Affiliated Hospital of Guangzhou University of Chinese Medicine, Guangzhou, Guangdong China; 3 Key Unit of Methodology in Clinical Research, Guangdong Provincial Hospital of Chinese Medicine, Guangzhou, Guangdong China; 4 Global Health - Health Systems and Policy (HSP): Medicines, focusing antibiotics, Department of Public Health Sciences, Karolinska Institutet, Stockholm, Sweden; 5 Clinical Research Center, South China Research Center for Acupuncture and Moxibustion, Medical College of Acu-Moxi and Rehabilitation, Guangzhou University of Chinese Medicine, Guangzhou, 510006 China

**Keywords:** Hyperlipidemia, Core outcome set, Chinese medicine, Systematic review, Delphi

## Abstract

**Background:**

Hyperlipidemia, defined as elevated lipid levels, is the primary and major risk factor for atherosclerotic cardiovascular disease. Several studies have evaluated the effects of Chinese medicine (CM) on hyperlipidemia. However, due to the varied designs and methods of these studies, data synthesis has been difficult, restricting the practical value of the findings. Developing a core outcome set (COS) could solve these methodological concerns. In this paper, we report a protocol to develop a COS for CM clinical trials for hyperlipidemia (COS-CM-Hyperlipidemia).

**Methods:**

The development of COS-CM-Hyperlipidemia will include four stages: (1) a systematic review to identify potential important outcomes—a study advisory group, composed of core stakeholders of hyperlipidemia, will be set up afterwards to evaluate the identified outcomes and a candidate outcome set will be developed accordingly; (2) a panel of experts will be invited to conduct a three-round Delphi survey, so that the experts’ opinions on the importance of outcomes for treating hyperlipidemia with CM will be collected; (3) a consensus meeting with clinicians, patients, and other key stakeholders will be conducted to finalize the items and definitions; and (4) COS-CM-Hyperlipidemia will be promoted and updated.

**Discussion:**

The development of this COS will improve the design and operation of CM trials on hyperlipidemia, keeping them in compliance with international standards, as well as the comparability and utility of their results.

**Trial registration:**

The Core Outcome Measures in Effectiveness Trials Initiative (COMET): http://www.comet-initiative.org/studies/details/983. Registered on 25 April 2017.

## Background

Hyperlipidemia, or elevated lipid levels, presents the symptoms of elevated total cholesterol (TC), low-density lipoprotein cholesterol (LDL-C), triglyceride (TG) levels, and mixed TC and TG [1]. It is a primary and major risk factor for atherosclerotic cardiovascular diseases, as well as a prerequisite for them [2–5]. In the United States, > 100 million adults have reported elevated TC and hyperlipidemia is the 11th leading cause for direct health expenditures ($37 billion annually) [6]. In China, the prevalence of hyperlipidemia is 34.0% and there is a significantly higher prevalence in men than women (41.9% and 32.5%, respectively) [7].

LDL-C has been recommended as the primary target of lipid-lowering interventions to reduce the incidence of atherosclerotic cardiovascular disease by the mainstream clinical guidelines [3, 4, 8]. In addition to conventional pharmaceutical therapies such as atorvastatin, Chinese medicine (CM) has abundant resources for lipid-lowering interventions. In CM theory, pattern (also called syndrome) is a diagnostic conclusion based on pathological changes in a disease at a certain stage. It includes features such as the nature of disease, cause, location, and development trends [9]. CM physicians treat patients according to syndrome differentiation, so it is important in clinical trials to measure syndrome change so as to embody the characteristics of CM [10].

Many studies have reported CM treatments that are effective against hyperlipidemia. For example, a meta-analysis indicated that the effect of Xuefuzhuyu decoction on hyperlipidemia was better than that in the control group [11]. Another systematic review showed that Yinchenwuling powder was more effective at decreasing the levels of TC and TG, while increasing the level of high-density lipoprotein cholesterol without serious adverse effects [12]. A Cochrane systematic review also found that Xuezhikang was the most commonly used herbal formula investigated for hypercholesterolemia; a significant effect on decreasing TC was shown in favor of Xuezhikang when compared with inositol nicotinate [13].

However, these promising findings need to be interpreted with caution due to methodological problems in CM trials [11–14]. Outcome measures are one of the key factors [15]. For instance, a systematic review of 35 trials studying the efficacy of a Chinese patented drug on hyperlipidemia reported as many as 30 different outcomes, but none focused on atherosclerotic cardiovascular disease events [16]. The main issues concerning hyperlipidemia outcome measures are: (1) the outcome measures vary from one trial to the next and this heterogeneity jeopardizes data consistency, resulting in reporting bias and patient irrelevance [17]; (2) there is no agreed upon or standardized outcome; (3) surrogate endpoints, such as biochemical indicators, have been widely adopted [11–16]; and (4) no standardized outcome sets to measure syndrome change for hyperlipidemia. Due to these problems, the practical value of clinical trials has been limited.

A core outcome set (COS) refers to a minimal set of important outcome measures identified by key stakeholders that should be collected and reported in all clinical trials of a specific health area, as a standard criterion [17, 18]. The Core Outcome Measures in Effectiveness Trials (COMET) Initiative brings together people interested in the development and application of COS. It collects and reports core outcomes that will promote the comparison and synthesis of separate trial results where appropriate [19]. Several studies on the development of COS for CM have already begun [20–22], but none focus on a COS for hyperlipidemia. This study presents a protocol for the development and promotion of a COS for hyperlipidemia in clinical trials. It evaluates the efficacy of CM in the treatment of hyperlipidemia compared with conventional medications. Therefore, a COS for CM clinical trials for hyperlipidemia is urgently need.

## Objectives

This study aims to present a protocol of the development of a COS for CM clinical trials for hyperlipidemia (COS-CM-Hyperlipidemia).

## Methods

**Fig. 1:**
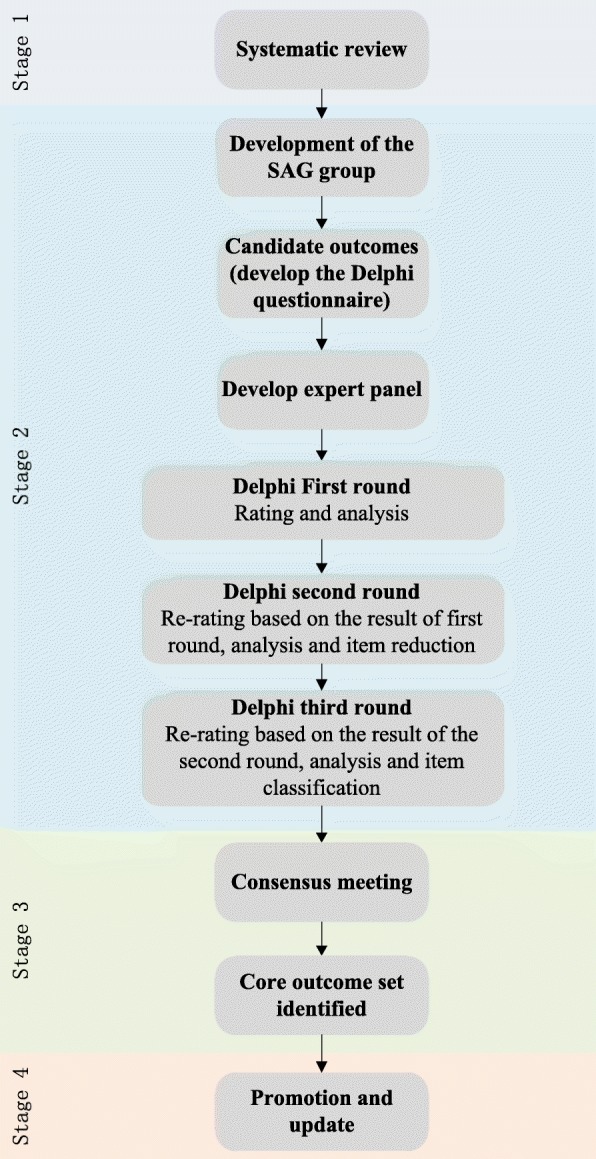
*Flow chart* of study design. *SAG* study advisory group

This study has been registered on the COMET website (number 983) [23]. The development of this COS-CM-Hyperlipidemia will include four stages: (1) a systematic review will be adopted to identify potential important outcomes—meanwhile, a study advisory group (SAG), composed of core hyperlipidemia stakeholders, will be set up to evaluate the identified outcomes and a candidate outcome set will be developed later; (2) a panel of experts will be selected and a three-round Delphi survey will be conducted to assess their opinions on the importance of hyperlipidemia outcomes in CM; (3) a consensus meeting, composed of clinicians, patients, and other key stakeholders, will be conducted to finalize the items and definitions; and (4) promotion and updates. Figure 1 illustrates the design of our study.

### Stage 1: Systematic review

A systematic review will be conducted to screen the range of potentially important outcomes according to the recommendations of the COMET Initiative [24]. Previous COS studies on CM have also used systematic reviews to initiate research [20–22]. All clinical trials (regardless of study type) that report hyperlipidemia outcome(s) will be included in this study.

#### Types of studies, interventions, and participants

Both interventional and observational studies (e.g. case reports, case-control studies, cohort studies, randomized controlled trials, and systematic reviews) published in either English or Chinese, will be included in our systematic review. Patients with hyperlipidemia, including hypercholesterolemia, hypertriglyceridemia, mixed hyperlipidemias, and lowered high-density lipoprotein cholesterol, treated by CM alone or combined with conventional medicine, will be considered for addition into our systematic review. Only those in which the hyperlipidemia diagnosis and the effectiveness assessment standard were clear will be eligible for inclusion. All patients, regardless of sex, age, or race, will be included. Patients who suffer from coronary heart disease, hypertension, diabetes, hypothyroidism, or liver disease will be excluded. If a study is mainly on secondary hyperlipidemia, as opposed to other serious diseases, the full text will be read to decide whether to include it.

#### Literature search

Data will be searched in CENTRAL, PubMed, Embase, Wangfang database, China National Knowledge Infrastructure, and Chinese BioMedical Database using a comprehensive, electronic search strategy. Two independent reviewers will assess the titles and abstracts to identify whether studies should be included. Any disagreements will be resolved through discussion after a thorough reading of the paper or by consulting a third researcher. Any duplicate studies will be excluded. Studies will also be excluded if they do not describe hyperlipidemia outcomes.

#### Data extraction and analysis

Two reviewers will independently extract the data by reading the full texts. For each included study, the following data will be extracted if available: author(s); year of publication; country; patient source; study type; treatment duration; length of follow-up; number of patients included; number of patients who withdrew; patients’ age; type of hyperlipidemia; CM syndrome types; interventions details; outcomes and their definitions; time-point; and method of outcome measurement. If any data are incomplete, the reviewers will contact the studies’ authors by email or telephone to get the missing data. Before the data are extracted, consistency evaluation will be conducted between the two reviewers to ensure all analytical details are reliable. Discussion and consulting with a third researcher will be conducted to resolve any disagreements. The data will then be entered into a form and the outcomes will be classified into different domains (patient-reported, clinician-reported, and proxy-reported outcomes) by one of our researchers. Classification will be confirmed by another researcher. The number of definitions of outcomes, the number of outcomes of each domain, and the number of measurement methods will be calculated and presented.

#### Study advisory group

A SAG composed of at least two hyperlipidemia patients and two clinicians, methodologists, researchers, ethicists, and statisticians will be created. The patients will be selected out of outpatients at the Guangdong Provincial Hospital of Chinese Medicine (GPHCM); clinicians will be selected from endocrinologists and cardiovascular experts of CM/integrated Chinese and Western medicine from both China and abroad; methodologists, researchers, and statisticians will be chosen from GPHCM and ethicists from GPHCM’s Ethics Committee.

#### Development of COS candidate items

The identified outcomes will be presented on a checklist which will be distributed to the SAG. The SAG will evaluate the checklist and domains of each outcome and will list additional outcomes if they think important ones have been left off the checklist. The final outcomes will be the candidate items for the COS in Questionnaire 1 and will be scored in Delphi process 1. The SAG will not participate in the Delphi process, but will take part in the consensus meeting.

### Stage 2: Delphi survey

#### Panel assembly

A wide variety of stakeholders, including hyperlipidemia patients, clinicians, methodologists, regulators, researchers, ethicists, statisticians, journal editors, and other relevant experts, will be recruited. There are no guidelines for the sample size of the Delphi study [25]; however, in general, the more panelists who participate, the more reliable the group judgment will be [26]. We therefore expect to select 50 panelists (including at least five patients and 10 experts) using “snowball sampling” (Table 1). The sample size will be based on the Construction of Core Outcome Set of Traditional Chinese Medicine Clinical Trials (COS-TCM) and the Implementation Specifications of the Delphi Method [27]. According to the principle of representativeness and authority, the experts will be selected from the following five professional fields: CM/integrated Chinese and Western medicine, clinical pharmacy, clinical epidemiology, statistics, and editors of important relevant journals. The identification of stakeholders will begin with a preliminary list of experts and patients. The preliminary patients will be selected from outpatients at GPHCM and the preliminary experts will be recognized authors of high impact papers and relevant senior physicians of the Chinese Association of Integrative Medicine. The preliminary list will then be augmented with authors of studies included in the systematic review. Finally, the experts and stakeholders will recommend whomever else they think should be included as a relevant stakeholder [25]. An email will be sent to the identified panelists who registered with informed consent to explain this study and the importance of the Delphi survey. Out of consideration for the response rate, we will email more related stakeholders than we plan to include. Each panelist will have a unique ID number corresponding to their contact information and their responses for each round of the Delphi survey.

**Table 1:** Stakeholder groups in the study

Stakeholder fields	Expected quantity	Recruitment methods
CM/integrated Chinese and Western medicine	15–20	(1) The identification of stakeholders begins with a preliminary list of experts and patients
Clinical pharmacy	4–6	(2) The preliminary list will be augmented with authors of studies included in the systematic review
Clinical epidemiology	4–6	
		(3) Stakeholders will recommend whomever else they think should be included as a relevant stakeholder
Statistics	4–6	
		(4) An email will be sent to the identified panelists who registered with informed consents to explain this study and the importance of the Delphi survey
Patients	15–20	
Editors of important relevant journals	4–6	Editors from important relevant journals on hyperlipidemia will be invited via email

#### Scoring method

Candidate items will be grouped by topic. In each group, candidate items will be sorted alphabetically. In this way, it will be convenient to both score items and avoid ranking bias in each group. For patient panelists, lay equivalents of each outcome will be presented instead of scientific terms [28]. Candidate items will be measured using a 9-point Likert scale, where 7, 8, and 9 mean “critical importance;” 4, 5, and 6 mean “not critically important;” and 1, 2, and 3 mean “low importance” [29]. This scale has been recommended by the COMET Initiative for measuring outcomes [24] and has been widely adopted by other COS development studies [17, 30–32].

#### Delphi round 1

In round 1 of the Delphi survey, all panelists will be asked to score all candidate items, along with their name, contact information, and other relevant personal information registered. This will confirm their stakeholder groups. After scoring all of the candidate outcomes, panelists will have the opportunity to add one or two additional outcomes they believe are important to hyperlipidemia but which had not been included in the questionnaire. A three-week period is planned for data collection and a reminder will be sent via email or SMS to panelists who have not completed their questionnaires by the end of the second week. A second reminder will be sent via email or SMS when only two days are left.

The number of panelists who complete Questionnaire 1 will be recorded. Each outcome will be analyzed using descriptive statistics, both by stakeholder group and as a whole. The SAG will assess the additional outcomes to decide whether they are representative of new outcomes. Those identified as new outcomes will be included in Delphi round 2. All scored outcomes will be included into Delphi round 2 (Questionnaire 2).

#### Delphi round 2

Panelists who complete the Delphi round 1 will be invited to take part in the Delphi round 2. They will re-score the outcomes listed on Questionnaire 2, where the scores they assigned in Delphi round 1 will be presented. The descriptive statistical analysis results of Delphi round 1, including the responses and distribution of each outcome from each different stakeholder group, will be attached. Panelists can make their scores in light of insight from others in his/her stakeholder group.

Descriptive statistics will be performed again. For each stakeholder group, we will calculate the number of participants and describe the distribution of each outcome score. A comparison will be conducted between each stakeholder group and the response group as a whole. Outcomes whose median is ≥ 4 (by any stakeholder group) will continue to Delphi round 3 (Questionnaire 3).

#### Delphi round 3

Panelists who complete Delphi rounds 1 and 2 will participate in round 3 to re-score Questionnaire 3. Questionnaire 3 will include those scores they gave in round 2. Descriptive statistical analysis of round 2, including the responses and distribution of each outcome, will be attached. Panelists will be asked to make their scores in light of the insight of all stakeholder groups.

After conducting the Delphi round 3 analysis, the outcomes will be categorized into three domains: “consensus out,” “consensus in,” or “without consensus,” using the definitions in Table 2. These prespecified definitions have also been suggested by the COMET initiative [24] and have been used by other COS development studies [33–36]. They can minimize the chance of consensus being defined post hoc and further decrease bias resulting from the pre-existing beliefs of the research team [37]. The consistency of these results across stakeholders, as well as across groups, will be analyzed. This analysis will ensure the relevance of outcomes across all participating stakeholders, making sure all voices are heard and that no minority stakeholders are suppressed [34]. The results of this process will be forwarded to the consensus meeting.

**Table 2:** Definition of consensus [35, 38]

Consensus classification	Description	Definition
Consensus out	Consensus that outcome should be excluded from the core outcome set	≥ 70% scoring 1–3 AND < 15% scoring 7–9 in each stakeholder group
Consensus in	Consensus that outcome should be included in the core outcome set	< 15% scoring 1–3 AND ≥ 70% scoring 7–9 in each stakeholder group
Without consensus	No consensus reached on whether the outcome should be included in or not	Other conditions

### Stage 3: Consensus meeting

#### Participants

To ensure a range of opinions of all hyperlipidemia stakeholder groups, the purposeful sampling method will be adopted to select participants from those who have completed all three rounds of the Delphi survey. The consensus group will be composed of at least two representatives from patients, endocrinologists, cardiologists, hyperlipidemia specialist nursing, and hyperlipidemia researchers, as well as all members of the SAG.

#### Process

A roundtable consensus meeting will be held at GPHCM to identify the final COS-CM-Hyperlipidemia. The results of each Delphi round and the classification of candidate outcomes from round 3 will be represented. Outcomes of “consensus in” will be voted either “yes” or “no” electronically and anonymously. Those voted for by at least 70% of the participants will be included in the final COS-CM-Hyperlipidemia. Outcomes scored “consensus out” will be excluded from the final COS-CM-Hyperlipidemia. Outcomes of “without consensus” in round 3 will be discussed. Participants will be asked to re-score all of the “without consensus” outcomes anonymously using the same 9-point Likert scale at the meeting. Outcomes for which at least 70% were scored 1–3 and at most 15% received a score of 7–9 will be removed from the final COS-CM-Hyperlipidemia. Those for which at least 70% were scored 7–9 and at most 15% were scored 1–3 will be included in the final COS-CM-Hyperlipidemia. The remaining outcomes will be re-scored until a final consensus is reached [28, 31].

### Stage 4: Promotion and update

A priority of this study is to improve the quality and value of CM trials for hyperlipidemia by applying the COS-CM-Hyperlipidemia. We will collaborate with systematic review groups, clinical guideline makers, clinicians, journal editors, and ethics committees to promote the broad application of COS-CM-Hyperlipidemia. We hope this COS-CM-Hyperlipidemia will be published in the C*OMET Initiative* and be recommended by relevant industry associations.

As medical research continues, understandings of disease, diagnosis, treatment, and evaluation will be updated. Therefore, the COS-CM-Hyperlipidemia needs constant evaluation and upgrading in accordance with the latest achievements of basic and clinical research. In the process of promotion, weaknesses in the current COS-CM-Hyperlipidemia should be noted. They should then be revised in line with the standard procedures based on the number and importance of these shortcomings. With constant evaluation and updates, the COS-CM-Hyperlipidemia can maintain its value and advantages in clinical and basic research of CM for hyperlipidemia. This is accomplished by adding new outcomes and new evaluation tools to ensure practicality and advancement.

## Discussion

To date, there has been no COS for CM clinical trials on hyperlipidemia. The development of COS-CM-Hyperlipidemia will improve the design and operation of CM trials, keeping them in compliance with international standards and guaranteeing the credibility of their results [15]. Outcomes important to key stakeholders, particularly to physicians and patients with hyperlipidemia, will be identified through the development of the COS-CM-Hyperlipidemia. We hope that the use of the developed COS-CM-Hyperlipidemia will ensure the consistency of important outcomes, lower reporting bias, improve the comparability of future studies, and improve the methodological quality of CM clinical research and the utility of study results.

## Study status

The development of the COS-CM-Hyperlipidemia is currently in the systematic review stage.

## Abbreviations

CM: Chinese medicine
COMET: Core Outcome Measures in Effectiveness Trials
COS: Core outcome set
COS-CM-Hyperlipidemia: Core outcome set of CM clinical trials for hyperlipidemia
GPHCM: Guangdong Provincial Hospital of Chinese Medicine
LDL-C: Low-density lipoprotein cholesterol
TC: Total cholesterol
TG: Total triglyceride
